# Differential Analysis of IAA Anabolism Pathway Based on *Bacillus cereus*, *Bacillus subtilis* and *Bacillus safensis* Genome

**DOI:** 10.1111/1758-2229.70323

**Published:** 2026-03-11

**Authors:** Dongyue Hou, Xuejie Zhai, Kezhong Zhang, Jinteng Cui

**Affiliations:** ^1^ College of Landscape Architecture, Beijing University of Agriculture Beijing China; ^2^ Beijing Laboratory of Urban and Rural Ecological Environment, Beijing University of Agriculture Beijing China; ^3^ Ancient Tree Health and Culture Engineering Technology Research Center, Beijing University of Agriculture Beijing China

**Keywords:** anabolism, bacillus, bacteria, genome, IAA

## Abstract

Bacteria of the genus Bacillus are widely used in agriculture due to their ability to synthesise IAA, but the metabolic pathways of IAA synthesis and the regulatory mechanisms of key genes remain unclear. In this study, the biochemical characteristics of *Bacillus cereus* , *Bacillus subtilis* and *Bacillus safensis* were detected. It was found that *Bacillus subtilis* has a higher indoles synthesis efficiency, *Bacillus safensis* has stronger salt tolerance and *Bacillus cereus* has more prominent alkali tolerance. Through whole‐genome sequencing and comparative analysis, combined with the retrieval of key genes in the tryptophan metabolic pathway and the metabolic analysis of indole compounds, it is clarified that *Bacillus cereus* has three IAA synthesis pathways, namely TAM, IAM and IPyA; *Bacillus subtilis* contains IAM and IPyA pathways; and *Bacillus safensis* only has the IPyA pathway. This study offers new insights into Bacillus metabolism, high‐quality strains for microbial agents, and matters for plant‐growth bacteria application and agricultural sustainability.

## Graphical abstract

Three Bacillus species isolated from *Platycladus orientalis* rhizosphere in Taihang Mountains: *Bacillus cereus* has 3 IAA synthesis pathways (TAM, IAM, IPyA); *Bacillus subtilis* has IAM/IPyA; and *Bacillus safensis* only has IPyA (via whole‐genome sequencing).

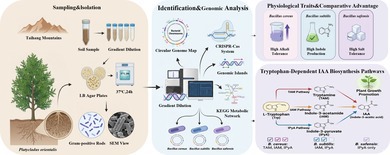

## 1 Foreword

As a core functional group of plant growth‐promoting rhizobacteria, Bacillus has irreplaceable niche value in the rhizosphere microecosystem. Most species of the genus Bacillus can remain in the vegetative state in nutrient‐replete pure culture systems and possess the biosynthetic capacity for indole‐like substances. Castillo‐Alfonso et al. (2022) conducted pure culture experiments using *Bacillus subtilis* as the model strain in modified M9 medium: when the strain utilised propionate or glucose as the carbon source and tryptophan as the co‐substrate, it could synthesise indole‐3‐acetic acid (IAA)—a key component of indole‐like substances—with yields reaching 310 and 230 mg/L, respectively. However, in natural soil and rhizosphere environments, Bacillus species are often induced to form dormant endospores under environmental stresses such as nutrient limitation. These spores are in a metabolically dormant and partially dehydrated state, unable to synthesise or secrete plant growth‐promoting metabolites (e.g., indole‐like substances), which significantly restricts the exertion of their plant growth‐promoting functions (McKenney et al. 2013). It is generally believed in domestic and international research that Bacillus has strong resistance to environmental stress, can produce various plant hormones and other substances that promote plant growth, can affect multiple characteristics of plants, and has potential in promoting plant growth (Dobrzyński et al. 2022; Kashyap et al. 2019). Studies have shown that IAA, as the main auxin in plants, plays an important regulatory role in the process of plant growth and development (Zhao 2010). As a hormone substance widely present in plants, IAA can affect the elongation and division of plant cells, the growth of primary and lateral roots and hypocotyls, the development of vascular tissues, as well as the formation of root hairs and floral organs (Woodward and Bartel 2005). It is of great significance to the growth and development of plants.

Currently, research on IAA production by the genus Bacillus is undergoing dual breakthroughs in cutting‐edge theories and technical methods. Significant progress has been made in such dimensions as the exploration of strain resources, the analysis of metabolic mechanisms, and the expansion of agricultural applications. Researchers continue isolating and identifying new Bacillus strains with the potential to produce IAA from niches such as soil and plant rhizosphere, such as *Bacillus mirabilis* YPR‐35 (Cai et al. 2024), *Bacillus thermopuppetii* MT (Zhou et al. 2023) etc. In addition to their high‐efficiency IAA synthesis ability, these strains also exhibit multiple growth‐promoting properties such as phosphorus dissolution, potassium release, siderophore production, and stress tolerance, providing high‐quality germplasm resources for the development of microbial inoculants (Shukla and Arnold 2025). Bacillus species mainly synthesise and metabolise IAA in vivo through the tryptophan‐dependent pathway. By means of molecular biology and multi‐omics techniques, the regulatory roles of some key genes in the IAA synthetic metabolic pathway have been preliminarily analysed, and the molecular mechanism of the tryptophan‐dependent pathway has been gradually clarified (Jiang et al. 2022), Such as the *ysnE* gene in *Bacillus amyloliquefaciens* SQR9 (Yang et al. 2025) encodes tryptophan acetyltransferase, catalyses the conversion of indolepyruvic acid and tryptamine to indole acetaldehyde, and regulates the conversion of indole‐3‐lactic acid to tryptophol, thus playing a key regulatory role in IAA synthesis and metabolism. The ability of Bacillus to synthesise and metabolise IAA has been widely applied in the development of microbial fertilisers, seed treatment agents, compost inoculants, etc., and has shown significant effects in promoting plant root development, increasing crop yield and improving soil fertility (Liu et al. 2022).

Nevertheless, there is still a lack of systematic analysis of the IAA synthetic metabolic pathways in Bacillus in this field. Therefore, in this experiment, whole genome sequencing and targeting metabolism analysis were performed on three strains of the genus Bacillus, namely *Bacillus cereus* , *Bacillus subtilis* and *Bacillus safensis* . The aim is to deeply explore the IAA synthetic metabolic pathways of these strains, reveal the molecular basis for their application, provide a theoretical basis for the practical application of Bacillus IAA synthesis and metabolism, and at the same time offer technical support for the preparation of multifunctional and high‐yield IAA Bacillus inoculants, thus laying an important foundation for the development and application of plant growth‐promoting bacteria.

## 2 Result

### 2.1 Morphological Characteristics of *Bacillus cereus* , *Bacillus subtilis* and *Bacillus safensis*

**FIGURE 1:**
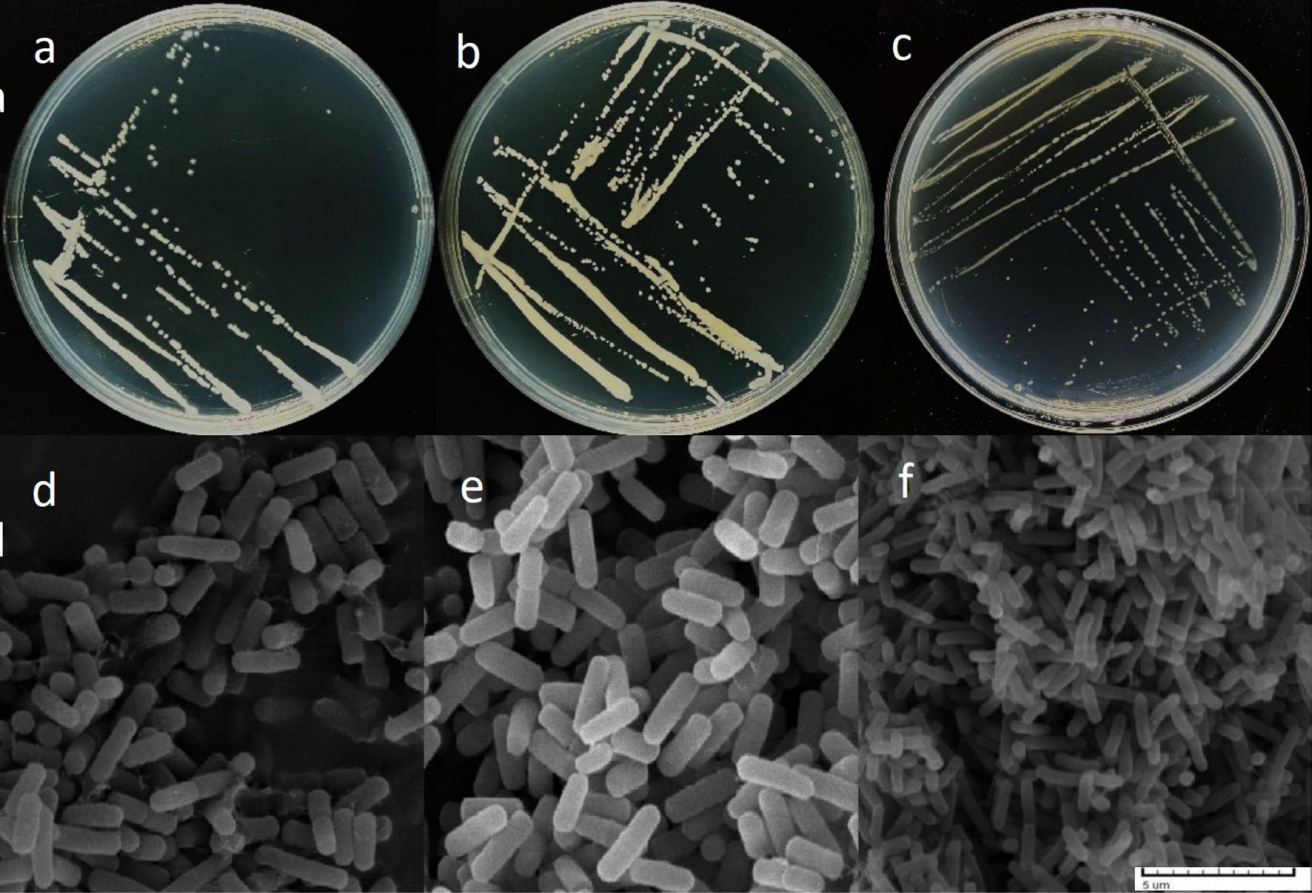
Morphological characteristics of *Bacillus cereus* , *Bacillus subtilis* and *Bacillus safensis* . (a) The isolation plate of *Bacillus cereus* ; (b) The isolation plate of *Bacillus subtilis* ; (c) The isolation plate of *Bacillus safensis* ; (d) The scanning electron microscope image of *Bacillus cereus* cells; (e) The scanning electron microscope image of *Bacillus subtilis* cells; (f) The scanning electron microscope image of *Bacillus safensis* cells. Bar = 5 μm.

We collected rhizosphere soil samples *of Platycladus orientalis* from the Taihang Mountains, and subjected the samples to serial dilution prior to spreading on LB agar medium. Following incubation at 37°Cfor 24 h, single colonies were purified via streak plate isolation (Figure 1a–c). Combined with Gram staining (Table 1) and colony phenotypic screening, three strains of presumptive Bacillus isolates were obtained.

**TABLE 1:** Functions of the selected strains.

Latin name	Indoles	Siderophore	Dissolve phosphorus	Potassium‐releasing	Producing protease	Colony growth under high salt concentration	Colony growth under alkaline conditions	pH	Gram staining
*Bacillus cereus*	+	−	−	+	+	0.508 ± 0.007b	0.625 ± 0.017a	6.72 ± 0.08b	+
*Bacillus subtilis*	+	−	−	−	−	0.308 ± 0.019c	0.285 ± 0.055c	7.10 ± 0.07a	+
*Bacillus safensis*	+	+	−	+	+	0.614 ± 0.016a	0.451 ± 0.004b	7.10 ± 0.07a	+

*Note:* ‘+’ indicates that the strain has this function, and ‘−’ indicates that the strain does not have this function. The values in the table are ‘mean ± standard deviation’. Values within the same column followed by different letters are significantly different (*p* < 0.05). Determination of colony growth under high salt concentration: 0.1 mL bacterial solution of salt‐tolerant strains obtained from preliminary screening was inoculated into LB liquid medium containing 0.5 mol/L NaCl. The control group was added with the same amount of sterile water, with nine repetitions set up. After 3 days of culture, the OD_600_ value was measured. The colony growth under high salt concentration in the control group was 0.000 ± 0.006. Determination of colony growth under alkaline conditions: 0.1 mL bacterial solution of alkali‐tolerant strains obtained from preliminary screening was inoculated into LB liquid medium (pH = 9). The control group was added with the same amount of sterile water, with nine repetitions set up. After 3 days of culture, the OD_600_ value was measured. The colony growth under alkaline conditions in the control group was 0.000 ± 0.002.

To resolve their taxonomic status, the isolates were cultured to the logarithmic growth phase, and their cellular morphology was visualised using a scanning electron microscope (SEM; JSM6360LV, JEOL, Japan), which preliminarily identified them as rod‐shaped bacteria. Subsequent whole‐genome sequencing analysis confirmed the three strains as *Bacillus cereus* , *Bacillus subtilis* and *Bacillus safensis* .

Microscopic observation results demonstrated that *Bacillus cereus* exhibited a bacillary morphology with square‐ended cells, measuring 1.0–1.2 μm in diameter and 3.0–5.0 μm in length (Figure 1d). *Bacillus subtilis* also presented a bacillary shape, but with rounded ends, with dimensions of 1.0–1.2 μm in diameter and 3.0–5.0 μm in length (Figure 1e). *Bacillus safensis* was characterised as bacilli with a diameter of 0.4–0.6 μm and a length of 1.5–3.7 μm (Figure 1f).

### 2.2 Functional Detection of *Bacillus cereus* , *Bacillus subtilis* and *Bacillus safensis* Strains

**FIGURE 2:**
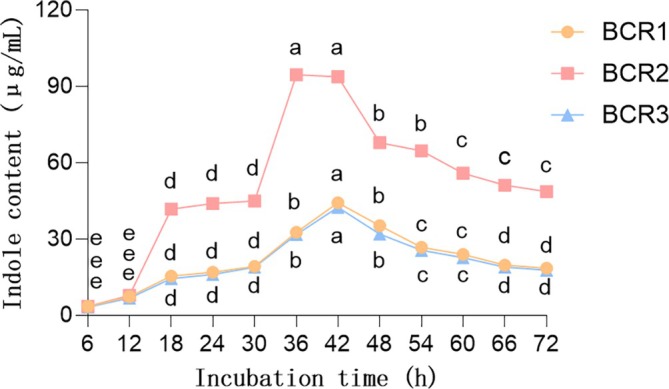
Effects of culture time on indole‐producing ability of strains. The ordinate represents IAA Content (μg/mL); the abscissa represents Culture Time (h). BCR1 is *Bacillus cereus* , BCR2 is *Bacillus subtilis* and BCR3 is *Bacillus safensis* . The strains BCR1, BCR2 and BCR3 were cultured in liquid LB medium for 72 h, followed by a comparative analysis of their indole‐like substance yields. The letters (a, b, c, d, e) above the data points indicate the results of the comparative analysis of indole‐like substance yields of the same strain at different culture time points. (*p* < 0.05, *n* = 9).

We used the Salkowski colorimetric method to identify the indoles synthesis and metabolism capabilities of the three strains. The identification results clearly show that all three strains have the ability to produce indoles (Figure S1), and the standard curve of indoles is shown separately (Figure S2). We detected the indoles content in the liquid medium of the three strains (Figure 2). When *Bacillus subtilis* was cultured for 36 h, the indoles content in the liquid medium reached the maximum value and then showed a downward trend with the extension of the culture time. The indoles synthesis and metabolism rates of *Bacillus cereus* and *Bacillus safensis* are slower than that of *Bacillus subtilis* . The indoles content in the liquid medium reaches the maximum when they are cultured for 42 h.

We detected the abilities of the three strains in siderophore production, phosphorus dissolution, potassium release, protease production, salt tolerance and alkali tolerance. The results showed that: the *Bacillus safensis* strain has the ability to produce siderophores (Figure S3); none of the three strains has the ability to dissolve phosphorus (Figure S4); both *Bacillus cereus* and *Bacillus safensis* strains have the ability to release potassium (Figure S5); both *Bacillus cereus* and *Bacillus safensis* strains have the ability to produce protease, with *Bacillus safensis* showing a stronger ability (Figure S6, Table S1); All three strains were capable of producing protease, with *Bacillus safensis* showing a stronger ability (Figure S6, Table S1). Additionally, all three strains exhibited salt tolerance (Figure S7), and *Bacillus safensis* achieved the highest growth under high salt concentration. When cultured in LB solid medium with an initial pH of 9, all three strains displayed alkali tolerance (Figure S8): the OD_600_ values of *Bacillus cereus* and *Bacillus safensis* exceeded 0.200, with the OD_600_ value of *Bacillus cereus* reaching 0.625, and the pH of the medium decreased from 9 to 6.72 during cultivation. Gram staining results confirmed that all three strains were Gram‐positive.

### 2.3 Basic Characteristics of Bacterial Genome and Prediction of CRISPR‐Cas

We performed whole‐genome sequencing on the strains of *Bacillus cereus* , *Bacillus subtilis* and *Bacillus safensis* . The sequencing results were screened, evaluated, and assembled and the basic genomic information of the strains was obtained (Figure S9). As can be seen from the sequencing results (Table 2), the genome size and total length of coding genes of the *Bacillus cereus* strain are the longest, being 5,786,006 and 4,798,041 bp, respectively; the *Bacillus subtilis* strain has the highest GC content, at 43.54%, and the longest average gene length, at 8107 bp. We used the CRTv1.2 software to predict CRISPR in the strains of *Bacillus cereus* , *Bacillus subtilis* and *Bacillus safensis* . The results showed that a total of 4 CRISPR‐Cas systems were predicted in the genome of *Bacillus cereus* ; a total of 9 CRISPR‐Cas systems were predicted in the genome of *Bacillus subtilis* ; and a total of 10 CRISPR‐Cas systems were predicted in the genome of *Bacillus safensis* . Detailed information is shown in Table S2.

**TABLE 2:** Basic characteristics of bacterial genomes.

	*Bacillus cereus*	*Bacillus subtilis*	*Bacillus safensis*
Genome size (bp)	5,786,006	4,217,970	3,663,256
GC content (%)	35.08	43.54	41.72
Number of reads	97,332	157,213	495,835
Average gene length (bp)	7591	8107	4686
CDS	5724	4237	3653
Total length of coding gene (bp)	4,798,041	3,715,563	3,217,800
Average length of coding gene (bp)	838	876	880
N50 (bp)	5,299,421	4,217,970	3,663,256
N50L	13,688	12,721	5271
tRNA	109	86	81
rRNA	42	30	24

### 2.4 Prediction of Genomic Islands

In the genome of *Bacillus cereus* , we predicted four genomic islands with a total length of 45,007 bp and an average length of 11,251.75 bp; a total of five genomic islands were predicted in the genome of *Bacillus subtilis* , with a total length of 246,440 bp and an average length of 49,288 bp; eight genomic islands were predicted in the genome of *Bacillus safensis* , with a total length of 95,379 bp and an average length of 11,922.375 bp. The distribution of genes in the genomic islands is shown in Table 3 (excluding hypothetical proteins).

**TABLE 3:** Results of genomic island prediction.

	Prophage ID	Initial position	Termination position	Length
*Bacillus cereus*	Island 1	949,016	956,686	7671
	Island 2	1,043,548	1,057,297	13,750
	Island 3	3,543,692	3,554,808	11,117
	Island 4	3,671,272	3,683,740	12,469
*Bacillus subtilis*	Island 1	31,056	157,036	125,981
	Island 2	1,852,855	1,888,962	36,108
	Island 3	2,753,666	2,759,302	5637
	Island 4	3,519,851	3,541,278	21,428
	Island 5	4,008,959	4,066,244	57,286
*Bacillus safensis*	Island 1	174,589	183,541	8953
	Island 2	696,556	705,820	9265
	Island 3	806,291	813,091	6801
	Island 4	918,225	924,896	6672
	Island 5	1,865,494	1,875,936	10,443
	Island 6	1,920,202	1,926,173	5972
	Island 7	2,321,191	2,362,478	41,288
	Island 8	3,147,523	3,153,507	5985

### 2.5 KEGG Annotation of Bacterial Genome

**FIGURE 3:**
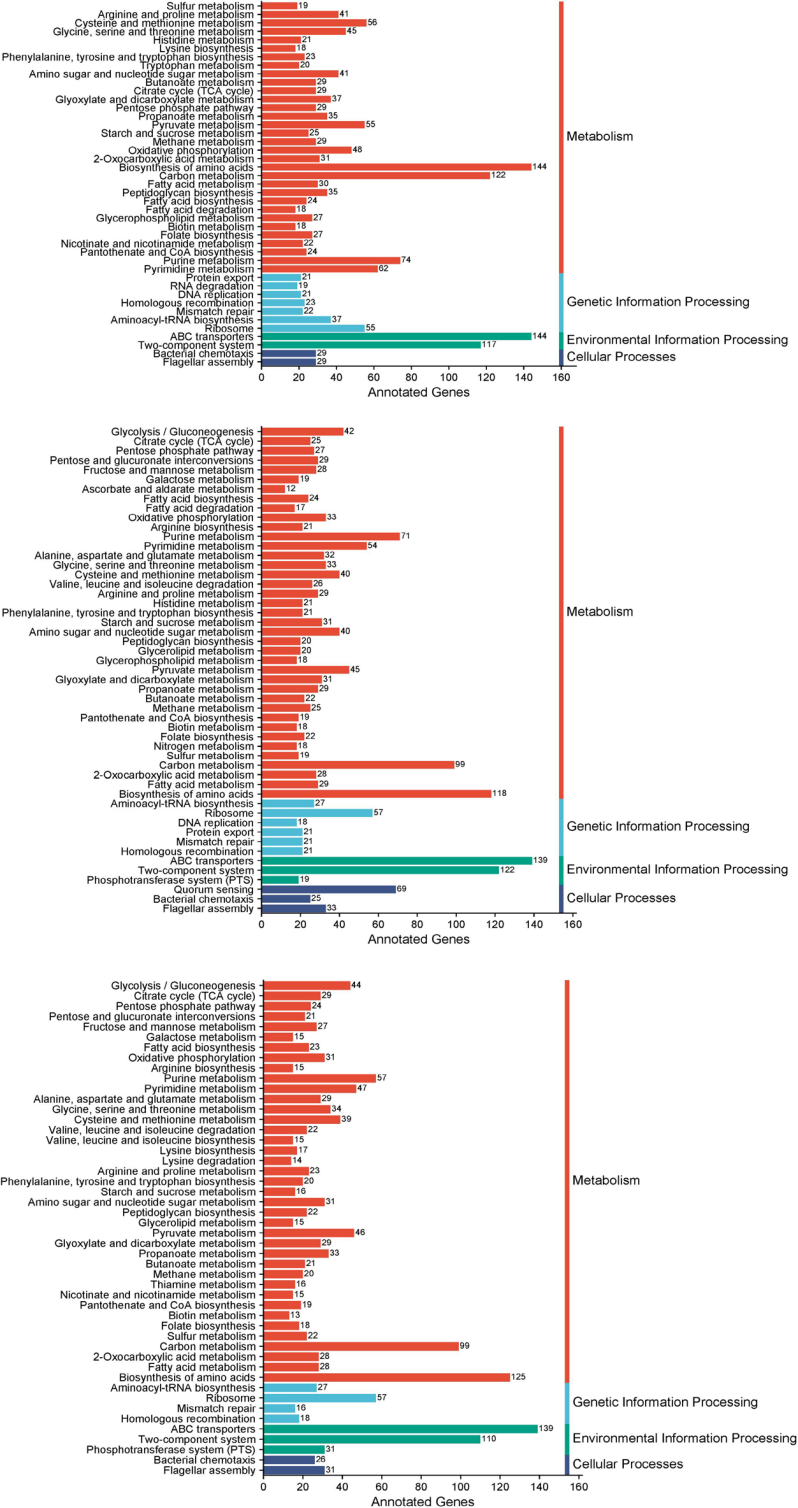
KEGG annotation map.

A total of 5708 genes were predicted from the genome of *Bacillus cereus* , among which 2491 genes were annotated with functional information based on the Kyoto Encyclopedia of Genes and Genomes (KEGG) database, accounting for 43.65% of the total predicted genes. According to the KEGG annotation results, 53.85% of the annotated genes were associated with metabolism, 10.47% with environmental information processing, 8.47% with genetic information processing, and 2.32% with cellular processes. For *Bacillus subtilis* , 4222 genes were predicted from its genome, with 2303 genes successfully annotated (54.54% of the total). Of these annotated genes, 52.71% were linked to metabolism, 13.06% to environmental information processing, 7.16% to genetic information processing and 3.82% to cellular processes. As for *Bacillus safensis* , 3467 genes were predicted from its genome, and 2086 genes were functionally annotated, representing 57.19% of the total predicted genes. The KEGG annotation showed that 53.21% of the annotated genes were involved in metabolism, 13.42% in environmental information processing, 6.47% in genetic information processing and 2.73% in cellular processes (Figure 3).

### 2.6 Analysis of Tryptophan Metabolic Pathway Based on Whole‐Genome Sequencing and Annotation

To analyse the tryptophan metabolic pathways in *Bacillus cereus* , *Bacillus subtilis* and *Bacillus safensis* , we selected homologous sequences of key enzymes involved in IAA biosynthesis—including tryptophan‐2‐monooxygenase, amidase, tryptophan aminotransferase, indolepyruvate decarboxylase, aldehyde dehydrogenase, tryptophan decarboxylase and monoamine oxidase—to perform BLAST searches against the genomic data of these strains, aiming to identify genes related to IAA anabolism. The results showed that a total of seven genes involved in auxin synthesis were annotated in *Bacillus cereus* ; a total of five differential genes involved in auxin synthesis were annotated in *Bacillus subtilis* ; and a total of three differential genes involved in auxin synthesis were annotated in *Bacillus safensis* (Table 4).

**TABLE 4:** Enzyme systems related to IAA metabolic pathways annotated in the whole genomes of *Bacillus cereus* , *Bacillus subtilis* and *Bacillus safensis* strains.

Pathway	Enzyme	Gene name	Name of bacteria	Gene ID	Length
Indole‐3‐acetamide (IAM)	Tryptophan‐2‐monooxygenase (EC: 1.13.12.3)	*iaaM*	*Bacillus cereus*	GE001827	1464 bp
			*Bacillus subtilis*	GE004097	1437 bp
			*Bacillus safensis*	—	—
	Amidase (EC: 3.5.1.4)	*ami*	*Bacillus cereus*	GE001731	1611 bp
			*Bacillus subtilis*	GE000615	780 bp
			*Bacillus safensis*	—	—
Indole‐3‐pyruvate (IPyA)	Tryptophan aminotransferase (EC:2.6.1.27)	*tat*	*Bacillus cereus*	GE003544	1434 bp
			*Bacillus subtilis*	GE001047	1014 bp
			*Bacillus safensis*	GE002461	1479 bp
	Indolepyruvate decarboxylase (EC: 4.1.1.74)	*ipd*	*Bacillus cereus*	GE002253	1677 bp
			*Bacillus subtilis*	GE003284	1725 bp
			*Bacillus safensis*	GE000023	1725 bp
	Aldehyde dehydrogenase (EC: 1.2.1.3)	*aldh*	*Bacillus cereus*	GE001171	1368 bp
			*Bacillus subtilis*	GE002295	1371 bp
			*Bacillus safensis*	GE000640	1485 bp
Tryptamine (TAM)	Tryptophan decarboxylase (EC: 4.1.1.28)	*tdc*	*Bacillus cereus*	GE002494	1455 bp
			*Bacillus subtilis*	—	—
			*Bacillus safensis*	—	—
	Monoamine oxidase (EC: 1.4.3.4)	*mao*	*Bacillus cereus*	GE001734	1437 bp
			*Bacillus subtilis*	—	—
			*Bacillus safensis*	—	—
	Aldehyde dehydrogenase (EC: 1.2.1.3)	*aldh*	*Bacillus cereus*	GE001171	1368 bp
			*Bacillus subtilis*	—	—
			*Bacillus safensis*	—	—

*Note:* — represents unannotated genes.

### 2.7 Determination of Indole Compounds in Fermentation Broth by UPLC–MS Method

Indole‐3‐acetamide, Indole‐3‐pyruvate, indole‐3‐acetonitrile, tryptamine, Indole‐3‐ethanol, Indole‐3‐lactic acid and indole‐3‐acetaldehyde are intermediate products of the Tryptophan‐dependent IAA synthesis metabolic pathway. Among them, indole‐3‐acetaldehyde is chemically unstable and can be converted into Indole‐3‐ethanol under certain conditions; indole‐3‐pyruvate can be converted to indole‐3‐acetamide under certain conditions.

We detected a total of 31 indole derivatives in the bacterial sludge and fermentation broth of *Bacillus cereus* , *Bacillus subtilis* and *Bacillus safensis* . Among them, 22 indole derivatives were detected in both the bacterial sludge and fermentation broth of *Bacillus cereus* ; 17 indole derivatives were detected in the bacterial sludge of *Bacillus subtilis* , and 19 in its fermentation broth; 18 indole derivatives were detected in the bacterial sludge of *Bacillus safensis* , and 19 in its fermentation broth (Table 5).

**TABLE 5:** Detection table of indole derivatives in bacterial sludge and fermentation broth of *Bacillus cereus* , *Bacillus subtilis* and *Bacillus safensis* .

Index	*Bacillus cereus*		*Bacillus subtilis*		*Bacillus safensis*	
	Bacterial sludge	Fermentation broth	Bacterial sludge	Fermentation broth	Bacterial sludge	Fermentation broth
Kynuric acid	+	+	+	+	+	+
Indole‐3‐acetic acid	+	+	+	+	+	+
Indole‐3‐acetaldehyde	+	+	+	+	+	+
l‐tryptophan	+	+	+	+	+	+
Indole‐3‐ethanol	+	+	+	+	+	+
Tryptamine	+	+	+	+	+	+
Indoxyl sulfate	+	+	+	+	+	+
Quinolinic acid	+	+	+	+	+	+
Nicotinic acid	+	+	+	+	+	+
N‐formylkynurenine	+	+	+	+	+	+
Picolinic acid	+	+	+	+	+	+
Anthranilic acid	+	+	+	+	+	+
Xanthine acid	+	+	+	+	+	+
L‐kynurenine	+	+	+	+	+	+
Indole‐3‐acetamide	+	+	+	+	+	+
N‐formylaminobenzoic acid	+	+	+	+	+	+
Indole‐3‐glyoxylic acid	+	+	+	+	+	+
O‐aminophenol	+	+	**−**	+	+	+
Indole‐3‐lactic acid	+	+	**−**	**−**	**−**	+
5‐methoxytryptamine	+	+	**−**	**−**	**−**	**−**
5‐Methoxyindole‐3‐acetic acid	+	+	**−**	+	**−**	**−**
3‐hydroxykynurenine	+	+	**−**	**−**	**−**	**−**
Indican	**−**	**−**	**−**	**−**	**−**	**−**
3‐Hydroxyanthranilic acid	**−**	**−**	**−**	**−**	**−**	**−**
2‐Amino‐3‐methoxybenzoic acid	**−**	**−**	**−**	**−**	**−**	**−**
Cinnabaric acid	**−**	**−**	**−**	**−**	**−**	**−**
2‐Ketoadipic acid	**−**	**−**	**−**	**−**	**−**	**−**
5‐Hydroxychromanol	**−**	**−**	**−**	**−**	**−**	**−**
Indole‐3‐acetonitrile	**−**	**−**	**−**	**−**	**−**	**−**
Serotonin	**−**	**−**	**−**	**−**	**−**	**−**
Melatonin	**−**	**−**	**−**	**−**	**−**	**−**
N‐acetyl‐5‐hydroxytryptamine	**−**	**−**	**−**	**−**	**−**	**−**
Indole‐3‐acrylic acid	**−**	**−**	**−**	**−**	**−**	**−**
Indole propionic acid	**−**	**−**	**−**	**−**	**−**	**−**
6‐hydroxy melatonin	**−**	**−**	**−**	**−**	**−**	**−**
N‐methylserotonine	**−**	**−**	**−**	**−**	**−**	**−**
5‐Hydroxyindole‐3‐acetic acid	**−**	**−**	**−**	**−**	**−**	**−**

*Note:* + represents the indole derivatives is annotated, − represents the indole derivatives is not annotated.

### 2.8 IAA Anabolic Pathway

**FIGURE 4:**
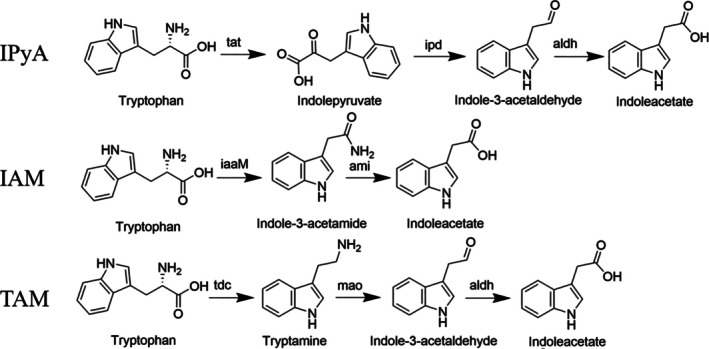
Predicted auxin synthesis pathways of *Bacillus cereus* , *Bacillus subtilis* and *Bacillus safensis* . *Bacillus cereus* harbours three indole‐3‐acetic acid (IAA) biosynthetic pathways, namely TAM, IAM and IPyA; *Bacillus subtilis* possesses two pathways, IAM and IPyA; while *Bacillus safensis* has only one pathway, IPyA.

**FIGURE 5:**
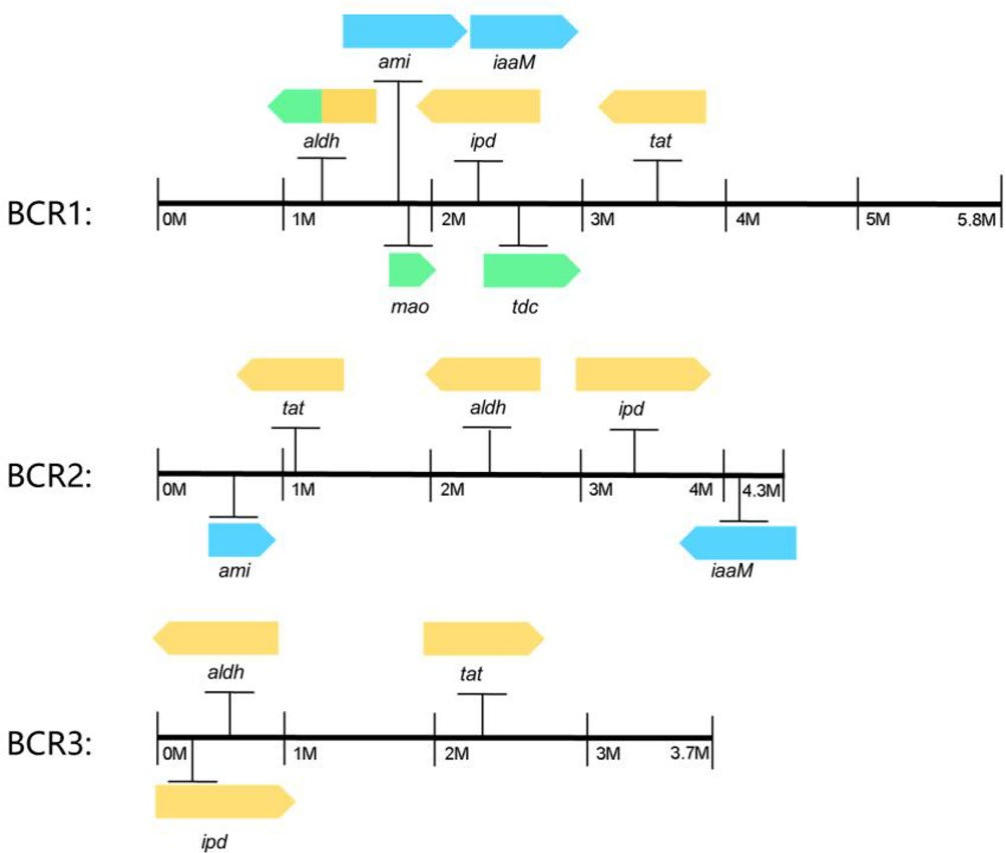
Gene locations of IAA metabolic pathway in the genomes of *Bacillus cereus* , *Bacillus subtilis* and *Bacillus safensis* . green, TAM pathway gene; orange, IPyA pathway gene; blue, IAM pathway gene.

Based on BLAST analysis and detection of indole derivatives, we analysed the IAA anabolism pathways of *Bacillus cereus* , *Bacillus subtilis* and *Bacillus safensis* (Figure 4). We hypothesise that there are three IAA anabolic pathways, namely TAM, IAM and IPyA, in *Bacillus cereus* . In the TAM pathway, monoamine oxidase catalyses the oxidation reaction of tryptamine; in the IAM pathway, amidase is involved in the hydrolysis process of indoleacetamide; in the IPyA pathway, aldehyde dehydrogenase catalyses the oxidation reaction of indole acetaldehyde. In contrast, *Bacillus subtilis* participates in the metabolic activities of the IAM and IPyA pathways through amidase and aldehyde dehydrogenase, while *Bacillus safensis* mainly relies on tryptophan aminotransferase, indolepyruvate decarboxylase and aldehyde dehydrogenase to construct the IPyA pathway. We marked the positions of key genes involved in the IAA anabolism pathway in the genomes of *Bacillus cereus* , *Bacillus subtilis* and *Bacillus safensis* (Figure 5).

## 3 Discussion

Many bacteria in the genus Bacillus can promote plant growth. The tryptophan‐dependent pathway is one of the major biosynthetic pathways for IAA synthesis, and the key genes of the tryptophan‐dependent pathway are the core factors regulating IAA anabolic metabolism (Li et al. 2024; Shao et al. 2015). As a core regulatory factor in plant growth and development, IAA is widely involved in plant stress responses, growth cycle regulation and physiological and biochemical processes (such as cell elongation and division, and vascular tissue differentiation) (Fu et al. 2015; Avsian‐Kretchmer et al. 2002), worth noting that the synthesis of IAA not only occurs in plants, but can also be accomplished by microorganisms symbiotic with them (such as those belonging to the genus Bacillus, other bacteria and fungi) (Spaepen et al. 2007). Plant roots can produce auxin precursor substances such as tryptamine, and these precursor substances can serve as substrates for IAA anabolic metabolism in bacteria, supporting microorganisms to generate IAA through the tryptophan pathway. This substrate‐product exchange between plant roots and microorganisms constructs a metabolic cycle of ‘plants synthesise precursors—microorganisms convert to IAA—plants utilise IAA’, which strengthens the synergistic relationship between plants and microorganisms in terms of auxin synthesis and signal regulation (He et al. 2021), promotes the improvement of plant growth and stress adaptability.

We conducted biochemical tests on *Bacillus cereus* , *Bacillus subtilis* and *Bacillus safensis* , and found that the strains of *Bacillus cereus* and *Bacillus safensis* have certain salt tolerance and the ability to synthesise and metabolise indoles. Among them, the salt tolerance of the *Bacillus safensis* strain is significantly higher than that of the other two strains, and the alkali tolerance of the *Bacillus cereus* strain is significantly higher than that of the other two strains. By setting up a time course and detecting the fermentation broths of *Bacillus cereus* , *Bacillus subtilis* and *Bacillus safensis* strains, it was found that the ability of *Bacillus cereus* , *Bacillus subtilis* and *Bacillus safensis* to produce indoles showed a trend of first increasing and then decreasing within 72 h, indicating that these bacteria all possess the relevant genes and metabolic pathways for IAA synthesis (Patten and Glick 1996). During the logarithmic growth phase of bacteria, when nutrients are sufficient and environmental conditions are favourable, these genes are activated and expressed, and the corresponding enzymes catalyse the synthesis of indoles, leading to a gradual increase in its content in bacterial cells (Spaepen et al. 2007). We hypothesise that as the extracellular accumulation of indole secreted by the cells reaches a certain concentration, it may trigger a feedback regulation mechanism. Indoles might inhibit the activity of key enzymes in the synthetic pathway or suppress the expression of related genes, thereby reducing indoles synthesis. Bacteria may also activate catabolic pathways to decompose excess indoles into other metabolites, leading to a decrease in its content (Wang et al. 2024).

In addition to the aforementioned metabolic regulatory mechanisms, bacteria also possess a unique adaptive immune defence mechanism, the CRISPR‐Cas system, which plays an important role in resisting the invasion of bacteriophages and exogenous plasmids (Barrangou 2015). In this study, we predicted that the genomes of *Bacillus cereus* , *Bacillus subtilis* and *Bacillus safensis* harbour 4, 9 and 10 CRISPR‐Cas systems, respectively. This is in some contrast to the findings of Zheng et al. (2020) study: in that study, among 1871 strains, only 13.9% of *Bacillus cereus* possessed CRISPR‐Cas systems, and most of these systems appeared to be defective based on gene content analysis. For this phenomenon, it is speculated that it may be related to the genetic background of the strains, ecological adaptability, and analytical methods. It may also be due to the diverse subtypes and complex sequence variations of the CRISPR‐Cas system, which may lead to false positive or false negative results (Barrangou and Marraffini 2014), thereby affecting the aforementioned statistical conclusions.

Genomic islands are discrete DNA fragments among closely related strains, and it is currently believed that their formation contributes to the diversification and adaptation of microorganisms (Juhas et al. 2009). Through the analysis of characteristics such as genomic GC content and tRNA loci, we predicted multiple possible genomic island regions. A total of four genomic islands were predicted in the genome of *Bacillus cereus* ; five genomic islands were predicted in the genome of *Bacillus subtilis* ; and eight genomic islands were predicted in the genome of *Bacillus safensis* . The difference in the number of genomic islands among different Bacillus species may reflect the unique adaptive strategies they have developed during long‐term evolution. Further analysis of their specific functions and impacts on bacterial phenotypes through expression analysis and experimental verification will provide important clues for in‐depth understanding of the adaptive evolutionary mechanisms of the genus Bacillus.

At present, the main pathways for bacterial anabolic metabolism of IAA have been clarified. They remove the amino group and carboxyl group from the α‐carbon of tryptophan through intermediates such as indolepyruvic acid, indoleacetamide or indole acetonitrile, and usually secrete the oxidised final product as IAA (Benítez et al. 2024). We searched for key genes of the Tryptophan‐dependent pathway in the genomes of *Bacillus cereus* , *Bacillus subtilis* and *Bacillus safensis* , and analysed the metabolites in the fermentation broths of *Bacillus cereus* , *Bacillus subtilis* and *Bacillus safensis* . It was found that the genome of *Bacillus cereus* was annotated with *iaaM*, *ami*, *tat*, *ipd*, *aldh*, *tac* and *mao*. Tryptamine, indole‐3‐acetamide and Indole‐3‐ethanol were detected in the fermentation broth. Based on the composition of key genes and metabolites, it is hypothesised that the *Bacillus cereus* strain may contain the TAM pathway, IAM pathway and IPyA pathway. The genome of *Bacillus subtilis* was annotated with *iaaM*, *ami*, *tat*, *ipd* and *aldh*, while *tac* and *mao* were not detected. Tryptamine, indole‐3‐acetamide and Indole‐3‐ethanol were detected in the fermentation broth, suggesting that the *Bacillus subtilis* strain may only contain the IAM pathway and the IPyA pathway. The genome of *Bacillus safensis* was annotated with *tat*, *ipd* and *aldh*, while *iaaM*, *ami*, *tac* and *mao* were not detected. Tryptamine, indole‐3‐acetamide and Indole‐3‐ethanol were detected in the fermentation broth, suggesting that the *Bacillus safensis* strain may only contain the IPyA pathway.

The study found that *Bacillus subtilis* has the highest efficiency in the synthetic metabolism of indoles. We compared the IAA synthetic metabolic pathways of *Bacillus subtilis* and *Bacillus safensis* , and found that the IAA synthetic metabolic pathway of *Bacillus subtilis* has an IAM pathway that is absent in *Bacillus safensis* . It is speculated that the IAM pathway, which can synthesise IAA through a two‐step catalytic reaction, is faster than the IPyA pathway that produces IAA through a three‐step catalytic reaction. Based on this, it is inferred that the IAM pathway in *Bacillus cereus* has the highest efficiency in the synthetic metabolism of IAA; comparing the IAA synthetic metabolic pathways of *Bacillus subtilis* and *Bacillus cereus* , it was found that *Bacillus cereus* also has an IAM pathway. However, the efficiency of IAA synthetic metabolism is not as high as that of *Bacillus subtilis* . It is speculated that *Bacillus cereus* synthesises and metabolises IAA through the TAM pathway and the IPyA pathway.

Plant roots secrete auxin precursor substances such as tryptamine, providing important substrates for the metabolic activities of rhizosphere microorganisms. *Bacillus cereus* , *Bacillus subtilis* and *Bacillus safensis* can utilise precursor substances such as tryptamine released by plant roots to synthesise IAA. This process constructs a metabolic cycle of ‘plant‐synthesised precursors—microbial transformation into IAA—plant utilisation of IAA’, realising the material circulation and energy flow between plants and microorganisms. When these Bacillus strains are applied by colonising around plant roots, the IAA synthesised and metabolised by them will effectively meet the demand for hormones in plant growth. This not only enhances the ability of roots to absorb water and nutrients, but also regulates the process of plant growth and development, improves the stress resistance of plants, thereby increasing plant yield and quality and providing powerful microbial resources for sustainable agricultural development.

## 4 Methods

### 4.1 Sampling, Isolation and Screening of Bacterial Strains

Samples were collected from the rhizosphere soil of *Platycladus orientalis* in the Taihang Mountains. For each sample plot, 10 g of sample was weighed and placed in a sterile conical flask containing 90 mL of sterile distilled water, followed by shaking at 180 rpm for 30 min. Subsequently, the sample suspension was subjected to gradient dilutions of 10, 100 and 1000 times, and 0.1 mL of each dilution was spread on sterilised LB solid medium (pH 7.0~7.2) (Sezonov et al. 2007). The plates were incubated at 30°C, and single colonies were picked for subculture for subsequent analysis. The strains were cultured in LB liquid medium, with time gradients set at 0 (control), 6, 12, 18, 24, 30, 36, 42, 48, 54, 60, 66 and 72 h, and nine replicates were set for each time gradient. The bacterial solution was cultured with shaking in a constant temperature shaker at 30°C and 180 rpm. At each time point, the bacterial solution was taken and centrifuged to obtain the supernatant, and the Salkowski colorimetric method was used to determine the indoles content (Gang et al. 2019). The OD values of *Bacillus cereus* , *Bacillus subtilis* and *Bacillus safensis* were measured at 530 nm, and their growth curves were plotted (Figure S2); the ability of strains to produce siderophores was evaluated using CAS solid medium (Louden et al. 2011); The phosphate‐solubilising performance of the strains was determined using the plate‐based detection method and phosphorus molybdenum blue colorimetric method (Nautiyal 1999); The potassium‐releasing ability of bacterial strains was determined using Aleksandrov medium containing bromothymol blue (Parmar and Sindhu 2018); The ability of strains to produce protease was detected using the skimmed milk agar plate method (Patil and Chaudhari 2008); The salt tolerance of the strains was evaluated using LB medium containing 0.5 mol/L sodium chloride (Microbe Notes 2024); The alkali tolerance of the strains was tested by culturing them on LB medium with pH = 9 (Sawatari and Yokota 2007).

### 4.2 Scanning Electron Microscope Observation

The morphology of bacteria was observed using a scanning electron microscope. Coverslips were inserted into the solid medium for culturing the bacterial strains. After the bacterial cells had adhered to the slips, the coverslips were removed. Subsequently, the precipitate was rinsed with phosphate buffer (pH 7.2), followed by the addition of 2.5% glutaraldehyde, and fixed at room temperature for 2–4 h. After fixation, the samples were placed in a refrigerator at 4°C overnight. After that, the samples were eluted with gradient ethanol (30%–95%) and then rinsed with tertiary‐butyl alcohol. After rinsing, 20 μL of tertiary‐butyl alcohol was added, and the mixture was placed in a refrigerator at −20°C until it froze and solidified. After treatment with critical point drying (HITACHI HCP‐2 critical point dryer) and gold sputter coating (Eiko IB‐3 ion coater), observations were made using a scanning electron microscope (SEM, JSM6360LV, JEOL, Japan) (Castillo et al. 2005).

### 4.3 Genome Sequencing and Functional Annotation

Genome sequencing was performed by Beijing Biomarker Technologies Co. Ltd. In the detection process, Thermo Fisher Scientific NANODROP2000 and Invitrogen Qubit3 Fluorometer were used to detect the nucleic acid concentration, and agarose gel electrophoresis was performed to evaluate the integrity. The library construction process was as follows: 2 μg of high‐quality nucleic acid was fragmented using G‐TUBE, then damage repair and end A‐tailing were completed with NEBNext FFPE DNA Repair Mix and NEBNext Ultra II End Repair/dA‐Tailing Module. Barcode sequences were added using the Nanopore EXP‐NBD104 (1–12 barcodes) kit, and sequencing adapters were ligated using EXP‐NBD114 (13–24 barcodes). For sequencing, the Flow cell Priming mix was prepared using the Nanopore EXP‐FLP001.PRO.6 chip preparation kit, and the on‐machine library was prepared with the SQK‐LSK109 ligation sequencing kit. Sequencing was performed on a PromethION48 sequencer equipped with a FLO‐PRO002 chip, using MinKnow software. In genome analysis, RepeatMasker v4.0.5 was used to identify repetitive sequences, CRT v1.2 was employed to detect CRISPR, IslandPath‐DIMOB v0.2 was applied to analyse genomic islands, Prodigal v2.6.3 was utilised for annotating coding genes and the KEGG database was used for functional gene classification (Bairoch and Apweiler 2000; Jensen et al. 2008; Kanehisa and Goto 2000; Finn et al. 2014).

### 4.4 Determination of Indole Compounds in Fermentation Broth

The detection of indole compounds in the fermentation broth was performed by Beijing Biomarker Technologies Co. Ltd. After the bacterial strains were cultured in liquid medium for 3 days, the supernatant of the medium and the bacterial cells were separated by centrifugation, followed by freeze‐drying treatment. An ExionLC AD ultra‐high performance liquid chromatograph coupled with a QTRAP 6500 + mass spectrometer system was used. The chromatographic separation conditions were as follows: a Waters ACQUITY UPLC HSS T3 C18 column (100 × 2.1 mm, 1.8 μm) was used, with mobile phase A being ultrapure water (containing 0.1% formic acid) and mobile phase B being acetonitrile (containing 0.1% formic acid). Tryptophan and its metabolites were analysed based on the scheduled multiple reaction monitoring (MRM) mode. The parameters of each MRM ion pair were determined by optimising the declustering potential (DP) and collision energy (CE), and data collection was performed using Analyst 1.6.3 software.

### 4.5 Statistical Analysis of Data

One‐way analysis of variance was performed on the data using IBM SPSS Statistics for Windows, version 27.0 (IBM Corp., Armonk, N.Y., USA) to assess the significance of the data (*p* < 0.05). Graphs were plotted using OriginLab Corporation, Origin, 2021 version (OriginLab Corporation, Northampton, Massachusetts, USA, 2021).

## Author Contributions

**Dongyue Hou:** writing – editing, writing – original draft, validation, software, formal analysis, data curation. **Xuejie Zhai:** investigation, data curation. **Kezhong Zhang:** writing – review and editing, project administration, funding acquisition, conceptualization. **Jinteng Cui:** writing – review and editing, project administration, funding acquisition, conceptualization.

## Ethics Statement

This article does not contain any studies with human participants or animals performed by any of the authors.

## Conflicts of Interest

The authors declare no conflicts of interest.

## Supporting information


**Data S1:** Supporting Information.

**Table S1:** Determination results of protease‐producing ability of strains.

**Table S2:** CRISPR prediction results.

## Data Availability

The raw reads of bacterial genome sequencing data (.fastq format) are deposited in the NCBI Sequence Read Archive (PRJNA1293753).
