# Supplementary material for: Differential Analysis of IAA Anabolism Pathway Based on Bacillus cereus, Bacillus subtilis and Bacillus safensis Genome
Source: Environ Microbiol Rep. 2026 Mar 11;18(2):e70323. doi: 10.1111/1758-2229.70323 (PMC13140672; doi:10.1111/1758-2229.70323)
Supplement: Supplementary file 1 — Data S1: Supporting Information. [file EMI4-18-e70323-s001.docx]

Supplement


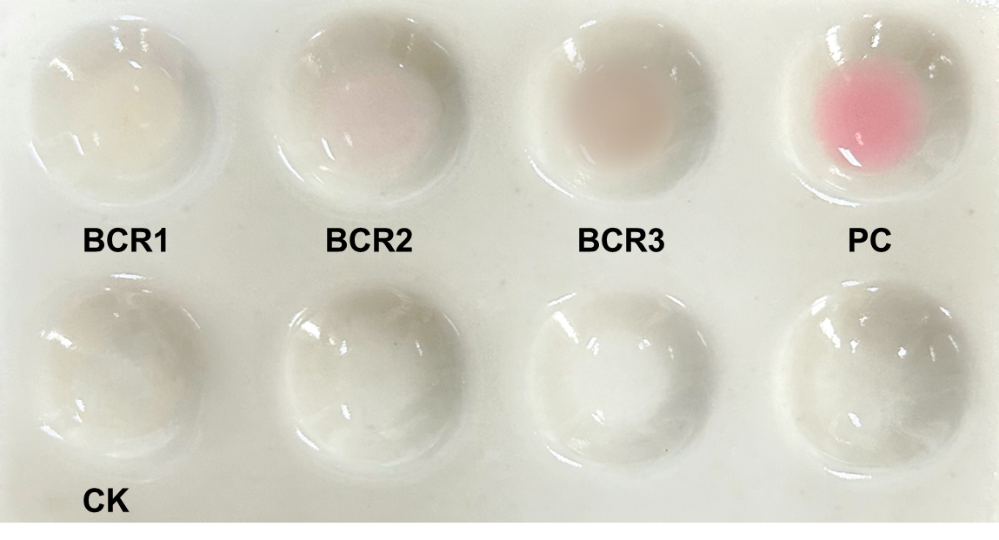


**Fig.S1** Qualitative detection results of indoles produced by bacteria

Note: BCR1 refers to *Bacillus cereus*, BCR2 refers to *Bacillus subtilis*, and BCR3 refers to *Bacillus safensis*. PC stands for positive control, and CK stands for blank control. The depth of the color of the reaction solution can reflect the indoles -producing ability of the strains; the darker the color, the stronger the IAA-producing ability.

**
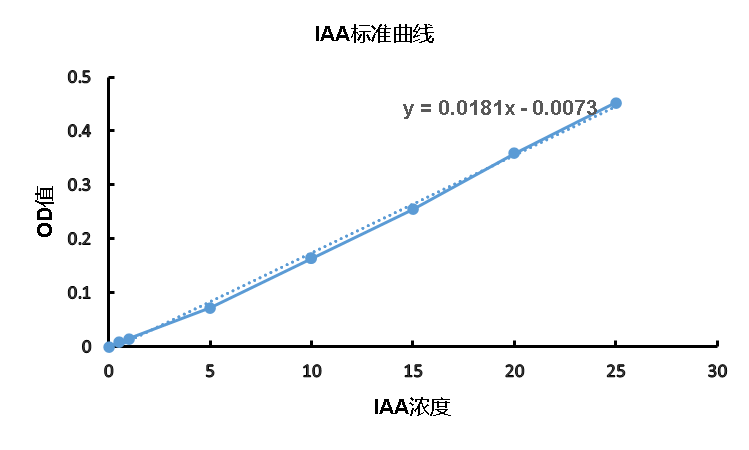
**

**Fig.S2** Indoles standard curve

Note: y represents the OD530 value, and x represents the indoles concentration.

**
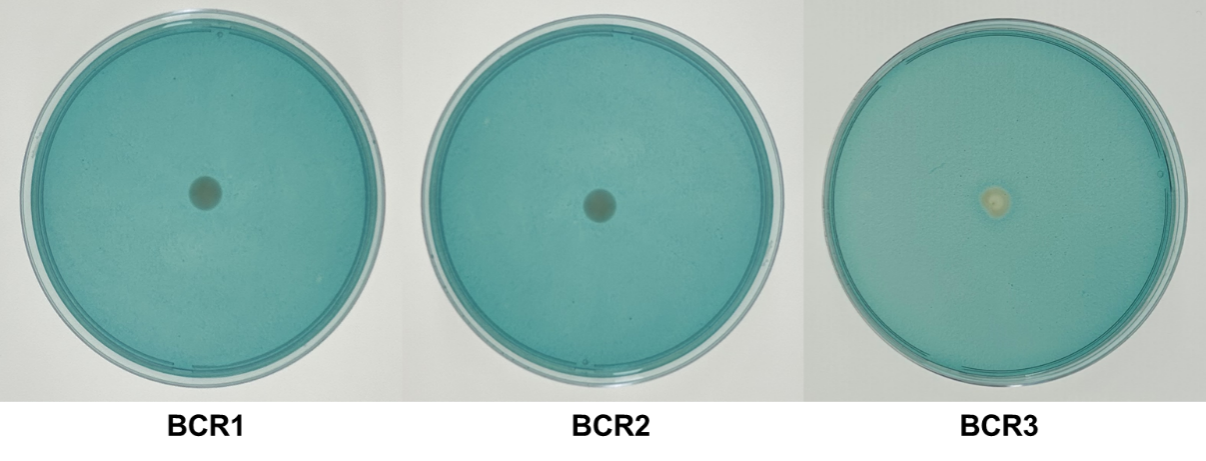
**

**Fig.S3** Screening results of siderophore-producing bacteria

Note: BCR1 refers to *Bacillus cereus*, BCR2 refers to *Bacillus subtilis*, and BCR3 refers to *Bacillus safensis*. When a strain has the ability to produce siderophores, an orange-yellow degradation circle will form around the colony. The color and size of the degradation circle can reflect the strength of the siderophore-producing ability.


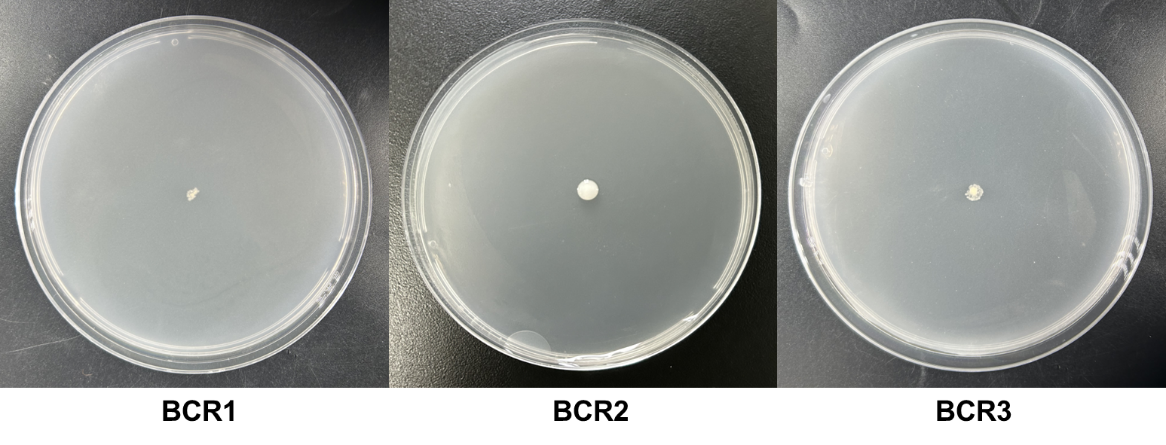


**Fig.S4** Results of phosphorus-dissolving bacteria

Note: BCR1 refers to *Bacillus cereus*, BCR2 refers to *Bacillus subtilis*, and BCR3 refers to *Bacillus safensis*. When a strain has the ability to dissolve phosphorus, a degradation circle will form around the colony. The color and size of the degradation circle can reflect the strength of the phosphorus-dissolving ability.


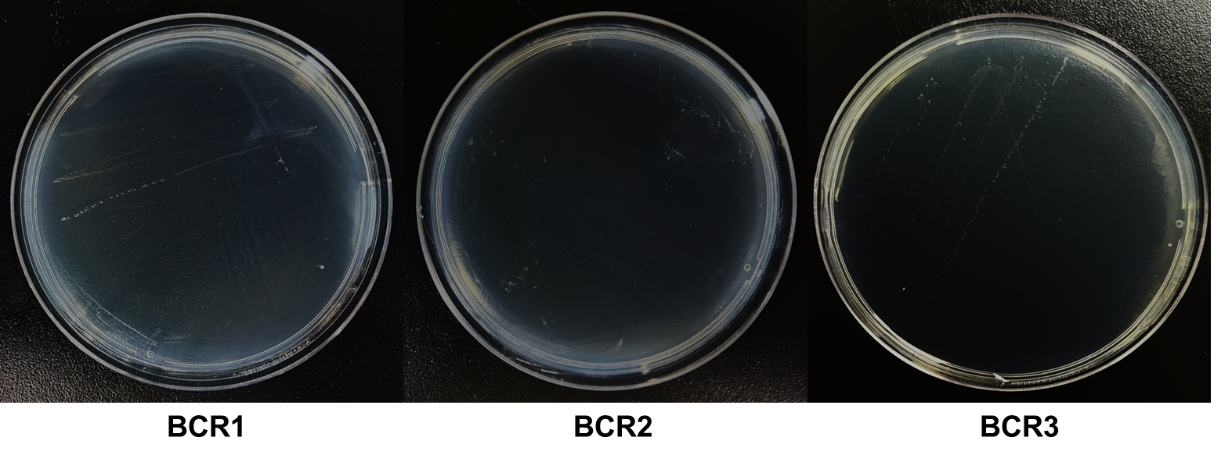


**Fig.S5** Results of potassium-releasing bacteria

Note: BCR1 refers to *Bacillus cereus*, BCR2 refers to *Bacillus subtilis*, and BCR3 refers to *Bacillus safensis*. Through three consecutive generations of screening, colonies that produce capsules and appear as smooth, transparent, and oil droplet-like were selected to determine whether the strain has a stable potassium-releasing ability. A total of 1 strain with potassium-releasing ability was obtained in the primary screening. Subsequently, the strain was re-inoculated onto silicate solid medium, and the three-zone streaking method was used for cultivation at 26°C for 2 days to observe the colony morphology.


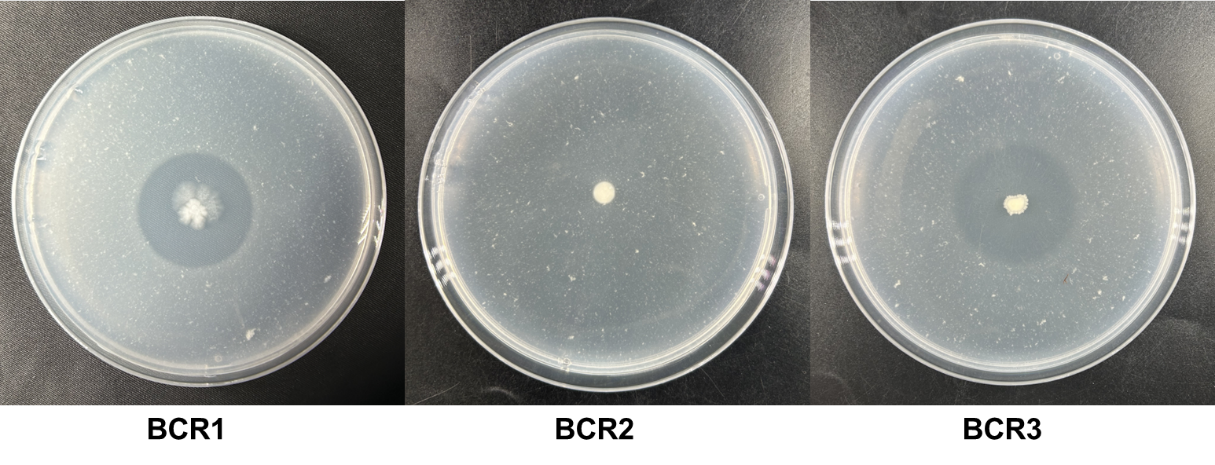


**Fig.S6** Screening results of protease-producing bacteria

Note: BCR1 refers to *Bacillus cereus*, BCR2 refers to *Bacillus subtilis*, and BCR3 refers to *Bacillus safensis*. When a strain has the ability to produce protease, a degradation circle will form around the colony. The color and size of the degradation circle can reflect the strength of the protease-producing ability.

Table S1 Determination results of protease-producing ability of strains

| Serial number | Strain number | Degradation circle (D)  (diameter/cm) | Strain (d)  (diameter/cm) | HC value  (D/d) |
| --- | --- | --- | --- | --- |
| 1 | BCR1 | 2.95 | 1.35 | 2.185 |
| 2 | BCR3 | 3.52 | 0.73 | 4.821 |

Note:BCR1 refers to *Bacillus cereus*,BCR3 refers to *Bacillus safensis*. HC value: the ratio of the diameter of the degradation circle to the diameter of the colony


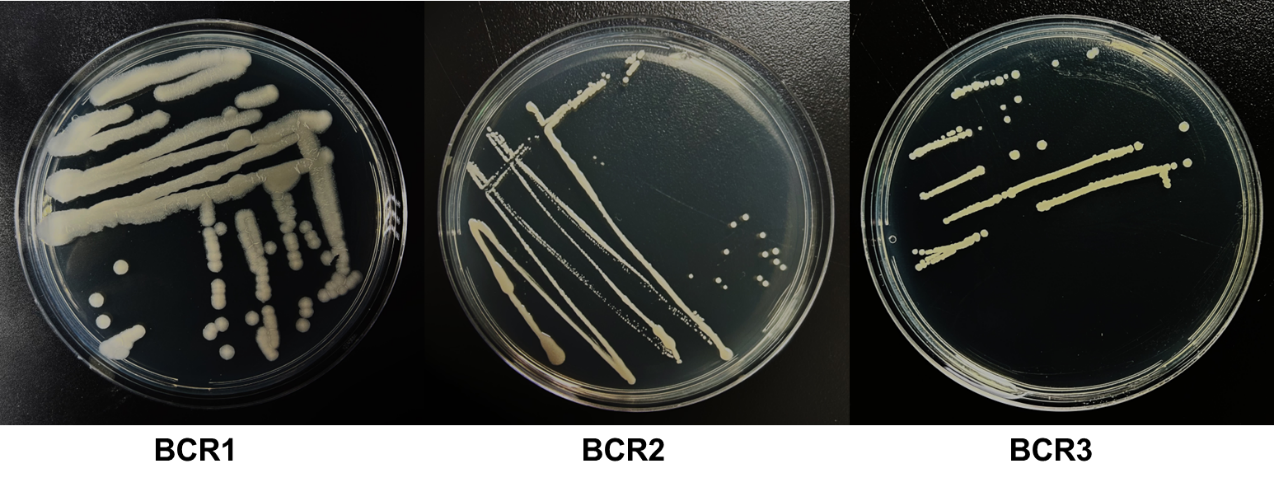


**Fig.S7** Results of salt-tolerant bacteria

Note: BCR1 refers to *Bacillus cereus*, BCR2 refers to *Bacillus subtilis*, and BCR3 refers to *Bacillus safensis*.


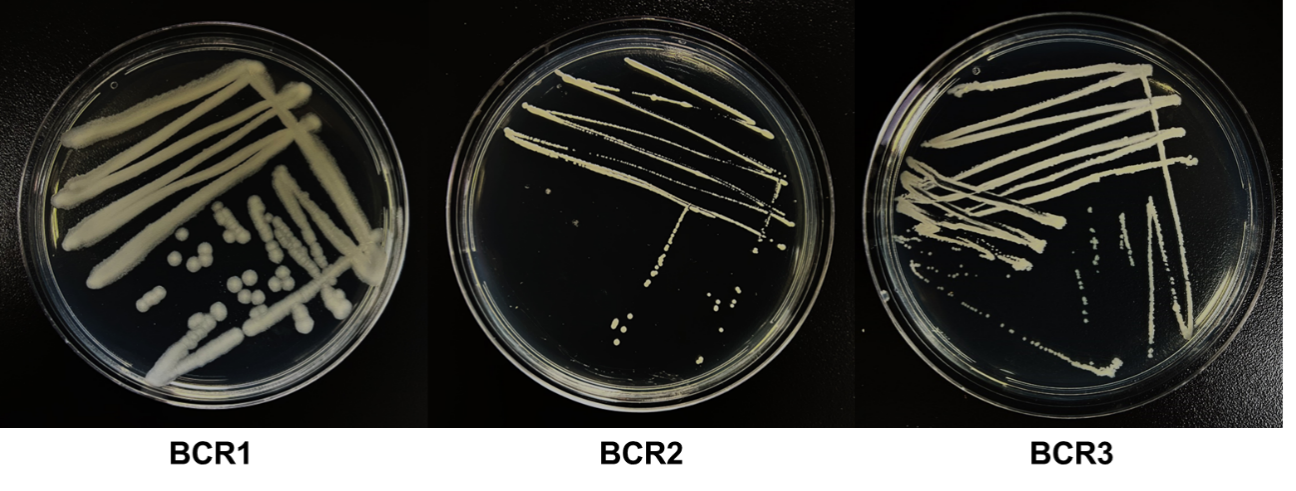


**Fig.S8** Results of kali-resistant bacteria

Note: BCR1 refers to *Bacillus cereus*, BCR2 refers to *Bacillus subtilis*, and BCR3 refers to *Bacillus safensis*.

Table S2 CRISPR prediction results

|  | CRISPR ID | Repeat number | Repeat sequence length | Intervening sequence | Length of interval sequence | Initial position | Termination position |
| --- | --- | --- | --- | --- | --- | --- | --- |
| *Bacillus cereus* | CRISPR1 | 12 | 20 | 11 | 36 | 433,454 | 434,094 |
|  | CRISPR2 | 11 | 23 | 10 | 19 | 535,615 | 536,057 |
|  | CRISPR3 | 7 | 21 | 6 | 45 | 790,447 | 790,863 |
|  | CRISPR4 | 5 | 26 | 4 | 26 | 901,635 | 901,870 |
| *Bacillus subtilis* | CRISPR1 | 2 | 27 | 1 | 26 | 109,744 | 109,823 |
|  | CRISPR2 | 4 | 32 | 3 | 49 | 697,722 | 697,996 |
|  | CRISPR3 | 16 | 26 | 15 | 55 | 697,668 | 698,908 |
|  | CRISPR4 | 3 | 23 | 2 | 25 | 1,243,628 | 1,243,746 |
|  | CRISPR5 | 2 | 19 | 1 | 47 | 1,276,413 | 1,276,497 |
|  | CRISPR6 | 5 | 22 | 4 | 38 | 1,512,082 | 1,512,343 |
|  | CRISPR7 | 9 | 20 | 8 | 22 | 2,499,127 | 2,499,482 |
|  | CRISPR8 | 3 | 32 | 2 | 49 | 2,501,735 | 2,501,928 |
|  | CRISPR9 | 2 | 19 | 1 | 26 | 2,501,927 | 2,501,990 |
| *Bacillus safensis* | CRISPR1 | 4 | 20 | 3 | 28 | 73,105 | 73,268 |
|  | CRISPR2 | 8 | 22 | 7 | 37 | 651,332 | 651,767 |
|  | CRISPR3 | 5 | 28 | 4 | 20 | 1,296,701 | 1,296,920 |
|  | CRISPR4 | 4 | 26 | 3 | 22 | 1,427,484 | 1,427,653 |
|  | CRISPR5 | 7 | 32 | 6 | 34 | 1,631,799 | 1,632,230 |
|  | CRISPR6 | 6 | 32 | 5 | 33 | 1,639,851 | 1,640,211 |
|  | CRISPR7 | 8 | 26 | 7 | 20 | 1,733,147 | 1,733,496 |
|  | CRISPR8 | 5 | 20 | 4 | 28 | 1,733,943 | 1,734,157 |
|  | CRISPR9 | 5 | 26 | 4 | 19 | 1,734,528 | 1,734,733 |
|  | CRISPR10 | 3 | 25 | 2 | 20 | 2,699,180 | 2,699,294 |


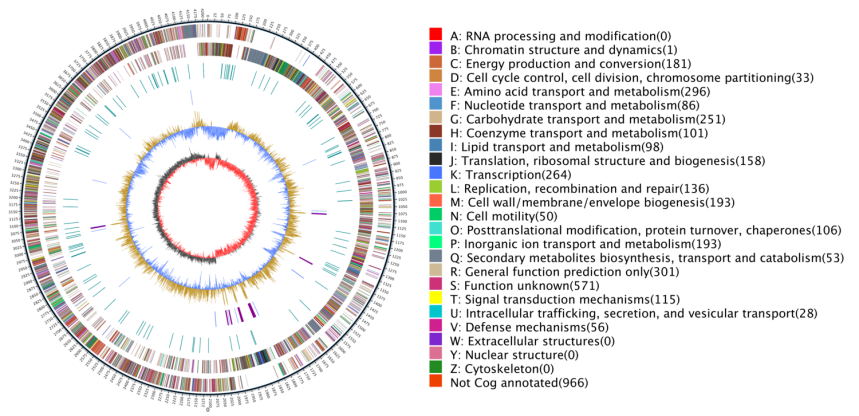

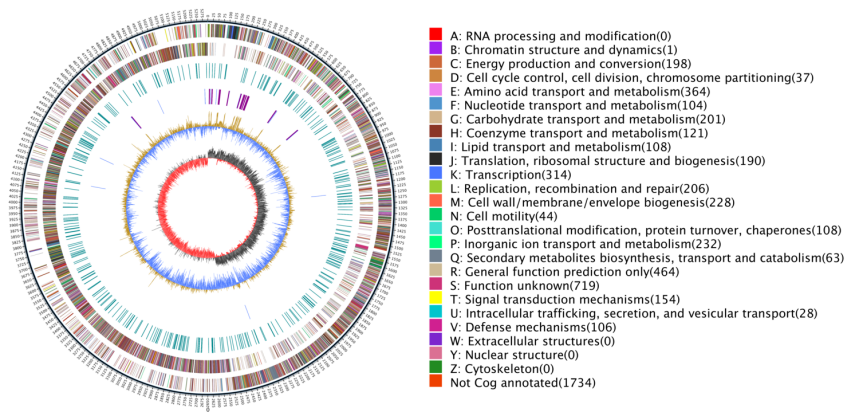

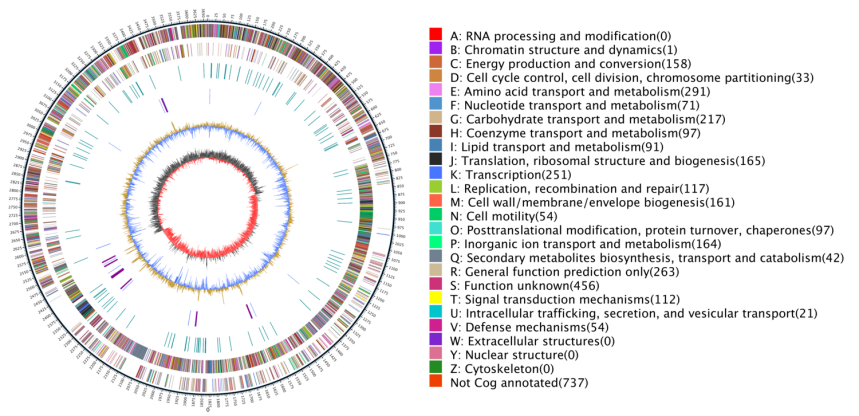


**Fig.S9** Genome map

Note:From top to bottom, the figures are the genome maps of *Bacillus cereus*, *Bacillus subtilis*, and *Bacillus safensis*, respectively.The outermost circle, with each scale representing 5kb, indicates the genome size. The second and third circles represent the genes on the positive and negative strands of the genome, respectively, where different colors correspond to different COG functional classifications. The fourth circle shows repetitive sequences. The fifth circle represents RNA and rRNA, with blue indicating tRNA and purple indicating RNA. The sixth circle displays the GC content: the light yellow parts indicate regions where the GC content is higher than the average GC content of the genome, and the higher the peak, the greater the difference from the average GC content; the blue parts indicate regions where the GC content is lower than the average GC content of the genome. The innermost circle is the GC-skew, where dark gray represents regions with a higher G content than C, and red represents regions with a higher C content than G.
